# Association between salivary microbiota and renal function in renal transplant patients during the perioperative period

**DOI:** 10.3389/fmicb.2023.1122101

**Published:** 2023-03-29

**Authors:** Xuyu Xiang, Bo Peng, Kai Liu, Tianyin Wang, Peng Ding, Hao Li, Yi Zhu, Yingzi Ming

**Affiliations:** ^1^The Transplantation Center of the Third Xiangya Hospital, Central South University, Changsha, China; ^2^Engineering and Technology Research Center for Transplantation Medicine of National Health Commission, Changsha, China

**Keywords:** salivary microbiota, renal function, renal transplantation, perioperative period, 16S rRNA

## Abstract

**Introduction:**

Renal transplantation is an effective treatment for the end stage renal disease (ESRD). However, how salivary microbiota changes during perioperative period of renal transplant recipients (RTRs) has not been elucidated.

**Methods:**

Five healthy controls and 11 RTRs who had good recovery were enrolled. Saliva samples were collected before surgery and at 1, 3, 7, and 14 days after surgery. 16S rRNA gene sequencing was performed.

**Results:**

There was no significant difference in the composition of salivary microbiota between ESRD patients and healthy controls. The salivary microbiota of RTRs showed higher operational taxonomic units (OTUs) amount and greater alpha and beta diversity than those of ESRD patients and healthy controls, but gradually stabilized over time. At the phylum level, the relative abundance of Actinobacteria, Tenericutes and Spirochaetes was about ten times different from ESRD patients or healthy controls for RTRs overall in time. The relative abundance of Bacteroidetes, Fusobacteria, Patescibacteria, Leptotrichiaceae and Streptococcaceae was correlated with serum creatinine (Scr) after renal transplantation.

**Discussion:**

In short, salivary microbiota community altered in the perioperative period of renal transplantation and certain species of salivary microbiota had the potential to be a biomarker of postoperative recovery.

## 1. Introduction

End-stage renal disease (ESRD) represents a serious public health problem fueled by aging populations and a pandemic of chronic non-communicable diseases, which is characterized by high mortality and economic burden. Renal transplantation is one of the effective treatments, with the hope of recovery for patients to normal life. However, there is still a lack of highly sensitive and specific biomarkers with minimal invasion and cost to assess recovery or rejection during the perioperative period.

The oral cavity consists of teeth, gingival groove, tongue, soft and hard palates, buccal mucosa, and tonsils. All the above areas are inhabited by microbiota and soaked in saliva all the time. Each salivary gland is highly permeable and surrounded by capillaries, a feature that allows for a freer exchange of substances between the salivary glands and blood (Wilson et al., 2014). Therefore, the salivary microbiota has the potential to be a bridge between oral (Belstrøm et al., 2018a) and systemic conditions.

Indeed, the relationship between chronic kidney disease (CKD) and gut microbiota has been widely investigated, both regarding changes in the floras of patients with CKD (Crespo-Salgado et al., 2016; Meijers et al., 2019; Ren et al., 2020) and regarding the mechanisms of gut microbiota in the development of CKD (Wang X. et al., 2020; Zhu et al., 2021; Wang et al., 2023). Saliva, one of the largest sources of gut microbiota, may play an important role in kidney disease that salivary microbiota ectopically colonizing the gut may be closely associated with the development of kidney disease and renal function. At the same time, several studies have discussed changes in salivary flora in patients with CKD (Hu et al., 2018; Duan et al., 2020; Guo et al., 2022; Liu et al., 2022). The overall composition of the salivary microbiota in CKD patients is significantly different from that of the healthy population, although the variation in individual flora or individual indicators is not entirely consistent across studies. Hence, the possibility of salivary microbiota functioning at CKD *in situ* cannot be ruled out. In summary, salivary microbiota has the potential as a diagnostic and therapeutic target for ESRDs or renal transplant recipients (RTRs).

Based on previous studies, we speculate that salivary microbiota in patients after renal transplantation will be significantly different from the preoperative flora and this change may be associated with renal function. Although the alteration of salivary floras in patients with ESRD has been studied, how salivary microbiota dynamic changes during the perioperative period of RTRs and the association between salivary microbiota and postoperative recovery have not been elucidated. Therefore, our study is the first to examine the variations of salivary microbiota during the perioperative period of renal transplantation and the relationship between salivary microbiota and renal function. We aimed to find some special floras associated with the return of renal function as clinical biomarkers.

## 2. Materials and methods

### 2.1. Subjects and sample collection

From 1 October 2022 to 18 October 2022, a total of 11 consecutive ESRD patients received renal transplantation in our center and were enrolled. Saliva samples were collected before surgery and at 1, 3, 7, and 14 days after surgery. Saliva samples from five healthy people were also collected as healthy controls. None of the above subjects had oral antibiotics, cortisol, smoking, or drinking history within 6 months.

Before collection, patients fasted for half an hour and rinsed their mouths. Patients spit the saliva into a sterile tube until it reaches 2 ml. Saliva was stored at −80°C immediately after collection.

The study protocol was approved (22207) by the Ethics Committee of the Third Xiangya Hospital of Central South University (Changsha, China). Written informed consent was obtained from all study participants. Experiments were carried out in accordance with the ethical guidelines set by the Declaration of Helsinki 1964 and its later amendments.

### 2.2. Sequencing

#### 2.2.1. Sampling and DNA extraction

Total genome DNA from samples was extracted using the CTAB/SDS method. DNA concentration and purity were monitored on 1% agarose gel. According to the concentration, DNA was diluted to 1 ng/μl using sterile water.

#### 2.2.2. Amplicon generation

16S rRNA genes were amplified using the specific primer 341F (CCTAYGG-GRBGCASCAG) and 806R (GGACTACNNGGGTATCTAAT) with the barcode. All PCR reactions were carried out in 30 μl of reactions with 15 μl of Phusion^®^ High-Fidelity PCR Master Mix (New England Biolabs); 0.2 μM of forward and reverse primers, and about 10 ng of template DNA. Thermal cycling consisted of initial denaturation at 98°C for 1 min, followed by 30 cycles of denaturation at 98°C for 10 s, annealing at 50°C for 30 s and elongation at 72°C for 60 s. And final extension at 72°C for 5 min.

#### 2.2.3. PCR products quantification and qualification

The same volume of 1X loading buffer (containing SYB green) with the PCR products and operate electrophoresis was mixed on a 2% agarose gel for detection. Samples with a bright main strip between 400 and 450 bp were chosen for further experiments.

#### 2.2.4. PCR products mixing and purification

PCR products were mixed in equidensity ratios. Then, the mixture of PCR products was purified with AxyPrep DNA Gel Extraction Kit (AXYGEN).

#### 2.2.5. Library preparation and sequencing

Sequencing libraries were generated using NEBNext^®^ Ultra™ DNA Library Prep Kit for Illumina (NEB, USA) following the manufacturer's recommendations, and index codes were added. The library quality was assessed on the Qubit^@^ 2.0 Fluorometer (Thermo Scientific) and Agilent Bioanalyzer 2100 system. Finally, the library was sequenced on an Illumina NovaSeq 6000 platform, and 250 bp paired-end reads were generated. Sequences are deposited under SRA PRJNA904953.

### 2.3. Data analysis

#### 2.3.1. OTU cluster and species annotation

Paired-end reads from the original DNA fragments were merged using FLASH. Sequences analysis was performed by the UPARSE software package using the UPARSE-OTU and UPARSE-OTUref algorithms. In-house Perl scripts were used to analyze alpha (within samples) and beta (among samples) diversity. Sequences with ≥97% similarity were assigned to the same OTU. We picked a representative sequence for each OTUs and used the RDP classifier to annotate taxonomic information for each representative sequence based on Silva 132 database.

#### 2.3.2. Phylogenetic distance and community distribution

Graphical representation of the relative abundance of bacterial diversity from phylum to species can be visualized using the Krona chart. Cluster analysis was preceded by principal component analysis (PCA), which was applied to reduce the dimension of the original variables using the QIIME software package. We used weighted UniFrac distance for principal coordinate analysis (PCoA) and Unweighted Pair Group Method with Arithmetic mean for the abbreviation (UPGMA) Clustering.

### 2.4. Statistical analysis

Linear discriminant analysis Effect Size (LEfSe) was used for the quantitative analysis of biomarkers within different groups. To identify differences in microbial communities between the two groups, ANOSIM and ADONIS were performed based on the Bray–Curtis dissimilarity distance matrices. A Wilcoxon rank-sum test and unpaired *t*-test were performed to evaluate differences between the two groups in alpha diversity, principal coordinates, and community difference analysis. Pearson correlation analysis was used to assess the correlation between microbiota and creatinine. A *p*-value of < 0.05 was required for the results to be considered statistically significant.

## 3. Results

### 3.1. Study population

The clinical information of RTRs (age 44.8 ± 12.2 years; 63.6% males) is shown in Table 1. The mean body weight index (BMI) was 20.5 ± 3.8 kg/m^2^ for RTRs. All RTRs received antihuman thymocyte globulin (ATG) for induction, the same triple immunosuppressive therapy, FK506, mycophenolate mofetil (MMF) plus steroids, and meropenem as a primary antibiotic. The saliva samples were collected before surgery (ESRD, *n* = 11) and at 1 day (RTR1, *n* = 9), 3 days (RTR3, *n* = 11), 7 days (RTR7, *n* = 11), and 14 days (RTR14, *n* = 8) after surgery. Generally, the specimens were divided into two groups, namely the ESRD group and the RTR group.

**Table 1:** Characteristics of renal transplant patients and control subjects.

**Patient**	**Gender**	**Age (years)**	**Height (m)**	**Weight (kg)**	**Body mass index (kg/m^2^)**	**Induction therapy**	**Immunosuppressive therapy**	**Antibiotics**	**Dialysis type**	**Dialysis duration (months)**	**Cause of end stage renal disease**	**Scr before KT (umol/L)**	**eGFR before KT (mL/min/1.73 m^2^)**	**Scr 7 days after KT (umol/L)**	**Scr 14 days after KT (umol/L)**
1	Female	45	1.58	51.5	20.63	ATG	FK506 + MMF +Steroids	Meropenem + Cefminox	HD	12	IgA Nephropathy	897	4.14	233	97
2	Male	53	1.62	67	25.53	ATG	FK506 + MMF +Steroids	Meropenem + Tikaolin	HD	8	Unknown	1,058	4.26	300	
3	Male	54	1.65	55.5	20.39	ATG	FK506 + MMF +Steroids	Meropenem + Carbofengin + Cefminol	HD	47	Unknown	753	6.37	204	136
4	Male	27	1.78	57.4	18.12	ATG	FK506 + MMF +Steroids	Meropenem + Colistin sulfate + Carbofengin+ Cephalosporin	HD	13	Unknown	601	10.12	238	103
5	Male	64	1.5	65	28.89	ATG	FK506 + MMF +Steroids	Meropenem + Peracillin	HD	6	Unknown	623	7.47	135	114
6	Female	51	1.67	48.7	17.46	ATG	FK506 + MMF +Steroids	Meropenem	PD	60	Unknown	1,016	3.42	87	95
7	Male	31	1.76	66.6	21.50	ATG	FK506 + MMF +Steroids	Meropenem+ Peracillin	PD	19	Unknown	1,104	4.72	132	104
8	Female	32	1.63	47	17.70	ATG	FK506 + MMF +Steroids	Meropenem	PD	13	IgA Nephropathy	974	4.11	73	78
9	Male	51	1.71	48.1	16.45	ATG	FK506 + MMF +Steroids	Meropenem + Tikaolin + Peracillin	HD	3	Unknown	1,231	3.59	109	132
10	Female	53	1.45	44	20.93	ATG	FK506 + MMF +Steroids	Meropenem + Cefminox	HD	12	Unknown	999	3.44	65	87
11	Male	32	1.75	55	17.96	ATG	FK506 + MMF +Steroids	Meropenem	HD	29	Unknown	1,556	3.09	124	98
**Control**	**Gender**	**Age (years)**	**Height (m)**	**Weight (kg)**	**Body mass index (kg/m** ^2^ **)**	**Scr (umol/L)**	**eGFR (mL/min/1.73 m** ^2^ **)**								
1	Female	50	1.57	48.5	19.68	73	212.54					
2	Female	41	1.52	63	27.27							
3	Male	56	1.5	49.3	21.91	49.3	313.91					
4	Male	30	1.65	69	25.34	90	98.39					
5	Male	32	1.87	90	25.74	73	316.2					

Healthy controls (HCs, *n* = 5) ranged in age from 30 to 56 years and consisted of three men and two women. The mean BMI was 24.0 ± 3.1 kg/m^2^, and the mean serum creatinine (Scr) was 71.3 ± 16.7 umol/L for HCs. We collected saliva samples (*n* = 9) from each of them two times, 7 days apart, to form the HC group. They all reported no history of chronic diseases or medication.

### 3.2. Impact of renal transplantation on salivary microbiota in individuals

First, according to the rarefaction curve, Shannon curve, and rank-abundance curve (Supplementary Figure 1), we found that the number of reads of most samples is reasonable. The curves tended to be flat, which indicated that the number of reads was relatively large enough to reflect species richness.

**Figure 1:**
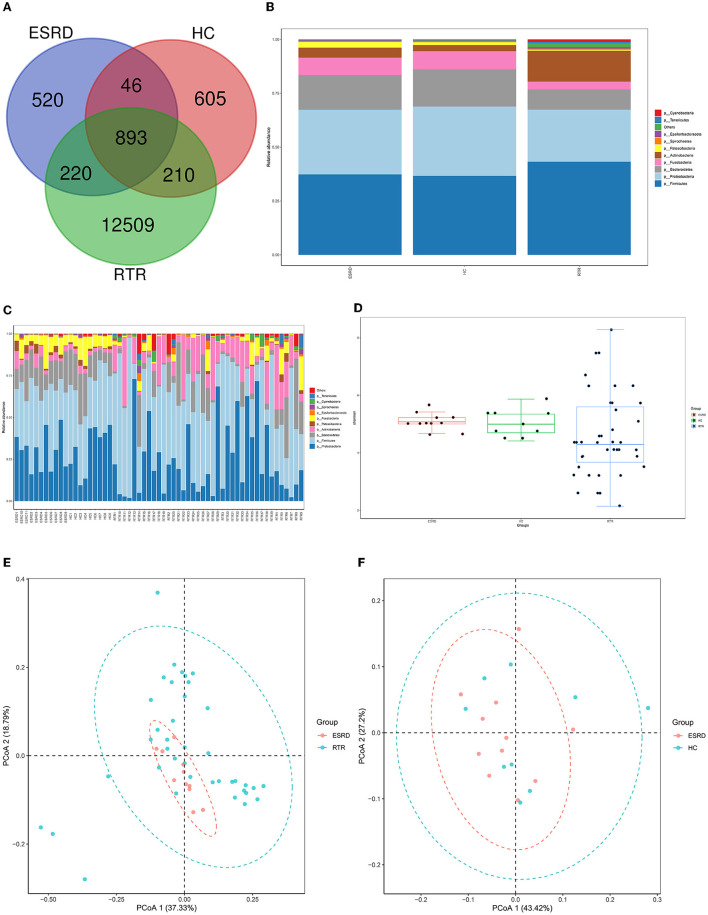
Composition characteristics of salivary microbiota in RTR, ESRD, and HC groups: **(A)** Venn graph for the OTUs of RTR, ESRD, and HC groups; **(B)** Salivary species composition of RTR, ESRD, and HC groups in the phylum level; **(C)** Salivary species composition of each individual in the phylum level; **(D)** Shannon index of RTR, ESRD, and HC groups; **(E)** PCoA graph of RTR and ESRD groups; **(F)** PCoA graph of ESRD and HC groups.

The Venn graph demonstrated the shared and unique OTUs between the three groups (Figure 1A). Overall, the RTR group has more OTUs. However, there was no significant difference in the number of OTUs in a single sample between the three groups. Figures 1B, C show the species composition of each group and individual samples at the phylum level. The ESRD and HC groups had relatively similar species composition, whereas the RTR group was quite different, especially in the relative abundance of Actinobacteria, Tenericutes, and Spirochaetes.

Alpha diversity, including Shannon, Simpson, and so on, provided a measurement of species diversity within a sample. The larger the Shannon index, the greater the diversity. The ESRD group was close to the HC group in alpha diversity. Group RTR always had larger intra-group differences (Figure 1D, RTR vs. ESRD: *p* < 0.05).

Beta diversity was used to study the intrinsic composition of the microbial structure. The closer the samples were to each other, the more similar the species' composition was. The PCoA was analyzed based on weighted UniFrac distance. According to the PCoA, the microbial composition of the RTR group was significantly different from those of the ESRD (Figure 1E) and HC (Supplementary Figure 2) groups, which was proved by the ADONIS analysis (RTR vs. ESRD: *p* = 0.001, RTR vs. HC: *p* = 0.001). On the contrary, the beta diversity of the ESRD group was not significantly different from that of the HC group (Figure 1F).

**Figure 2:**
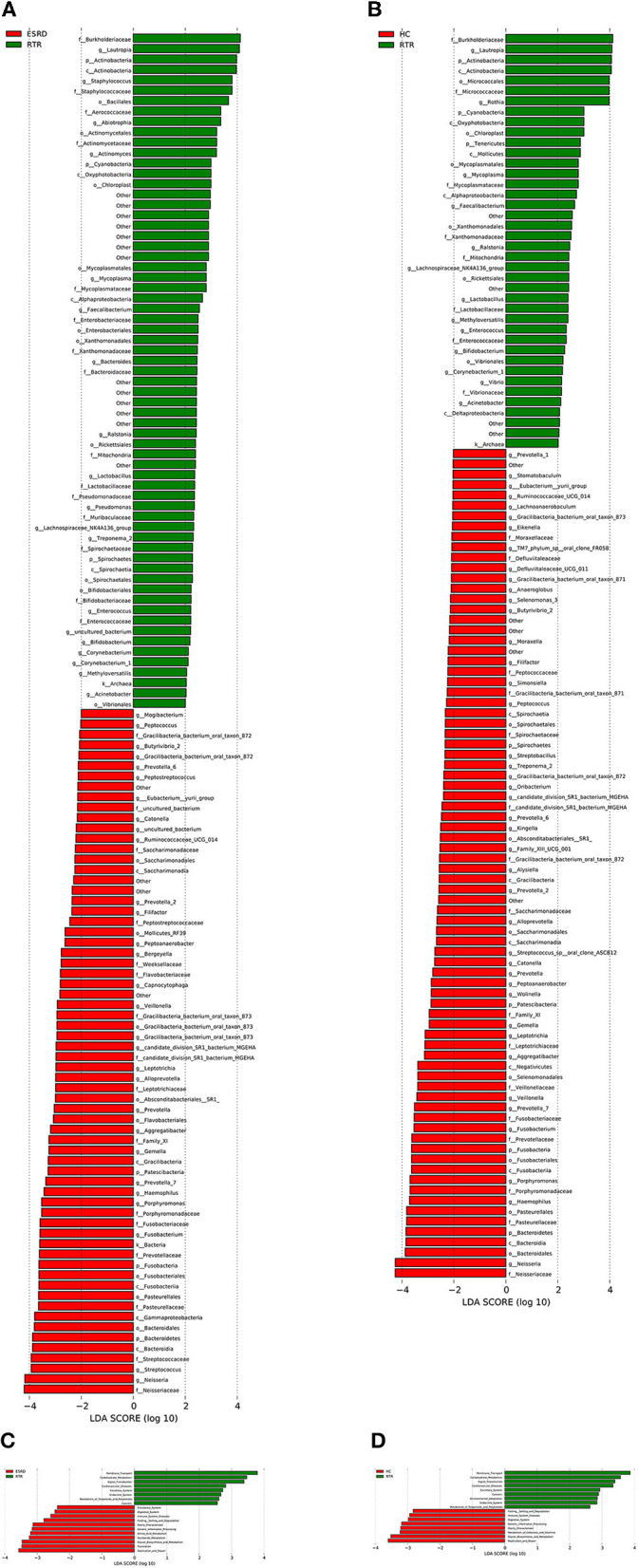
Differential species and KEGG analysis for RTR, ESRD, and HC groups: **(A)** LEfSe analysis of the salivary microbiota composition between RTR and ESRD groups; **(B)** LEfSe analysis of the salivary microbiota composition between RTR and HC groups; **(C)** LEfSe analysis of the predicted function between RTR and ESRD groups; **(D)** LEfSe analysis of the predicted function between RTR and HC groups.

LEfSe analysis was used to describe differential species between different groups. When the LDA value was >2, the species was a statistically significant biomarker between groups. The results showed that when compared with the ESRD (Figure 2A) and HC (Figure 2B) groups, the relative abundances of Burkholderiaceae, Lautropia, and Actinobacteria of the RTR group were significantly increased, and Neisseriaceae and Neisseria were significantly decreased. The composition of OTU sequences was further transformed into KEGG orthodontics to analyze the differences in predicted function. The pathways related to membrane transport, carbohydrate metabolism, and signal transduction were significantly enriched in the RTR group (Figures 2C, D).

### 3.3. The dynamic change in salivary microbiota during the early stage post-renal transplantation

**Figure 3:**
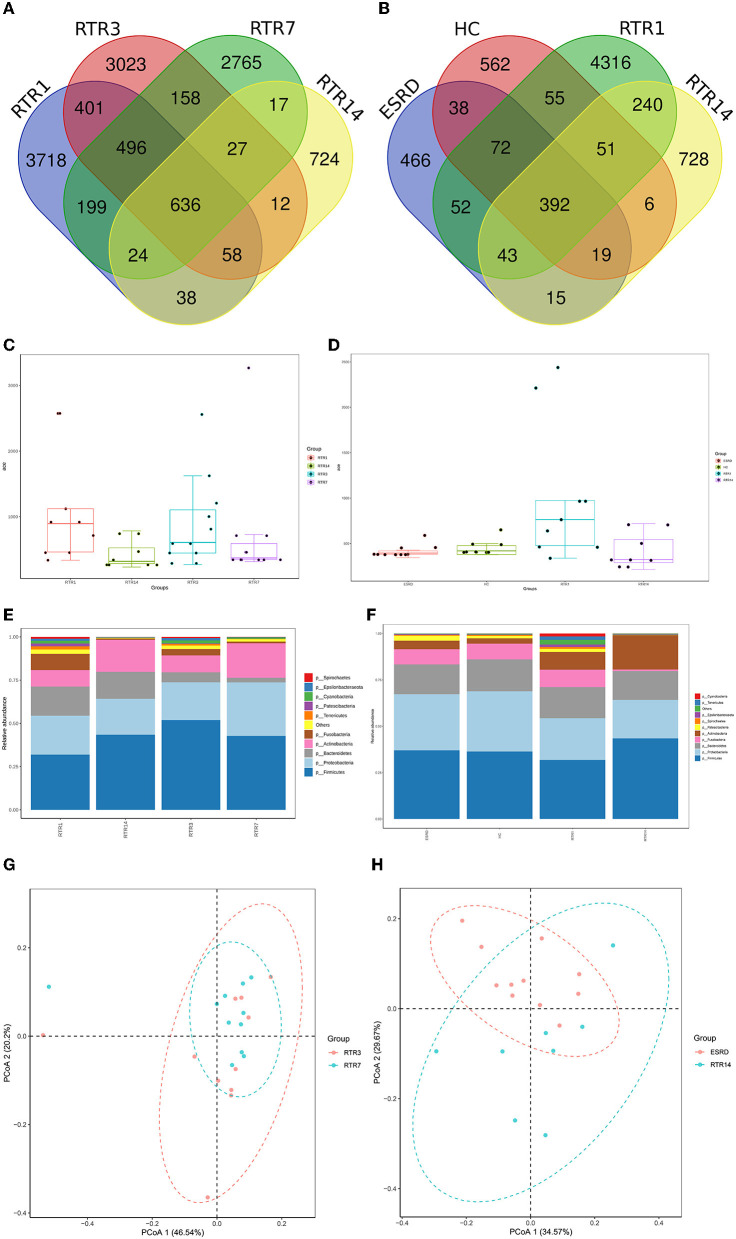
Composition characteristics of salivary microbiota at different time points and states: **(A)** the Venn graph for OTUs of the RTR group at different time points; **(B)** the Venn graph for OTUs of the RTR1, RTR14, ESRD, and HC; **(C)** the Ace index of the RTR group at different time points; **(D)** the Ace index of the RTR1, RTR14, ESRD, and HC; **(E)** salivary species composition of RTR group at different time points at the phylum level; **(F)** salivary species composition of RTR1, RTR14, ESRD, and HC at the phylum level; **(G)** PCoA graph of RTR3 and RTR7; **(H)** PCoA graph of RTR14 and ESRD.

The unique community occupied most OTUs of the RTR1, RTR3, and RTR7 and differed between every two adjacent time points (Figure 3A, RTR1 vs. RTR14: *p* < 0.05). Over time, the OTUs of RTRs gradually decreased and the shared OTUs with ESRD or HC groups increased, and RTR1 or RTR7 was significantly different from ESRD or HC (Figure 3B, *p* < 0.05). Figures 3C, D show that the Ace index and intra-group differences of the RTR group descended and approached ESRD and HC groups over time (RTR1 vs. ESRD: *p* < 0.05, RTR1 vs. RTR14: *p* < 0.05). Other alpha diversity indexes also showed the same changes in the salivary microbiota of RTRs at different time points (Supplementary Figure 3). At the phylum level, the microbial composition of the RTR group was constantly changing (Figure 3E) but always differed from ESRD or HC groups (Figure 3F). From PCoA, the intrinsic microbial composition of RTR1 and RTR14 groups significantly differed (*p* = 0.001) from ESRD or HC groups (Figure 3H and Supplementary Figure 4) and the intrinsic microbial composition significantly changed between 3 and 7 days after surgery (Figure 3G and Supplementary Figure 4).

### 3.4. Certain species of salivary microbiota were associated with the recovery of renal function

**Figure 4:**
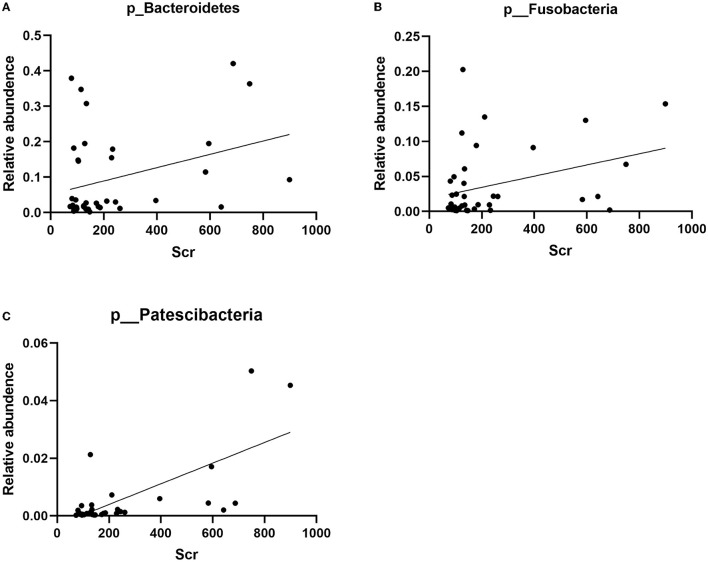
Correlation between Bacteroidetes, Fusobacteria, Patescibacteria, and Scr: **(A)** the scatter diagram of relative abundance of Bacteroidetes and Scr concentration; **(B)** the scatter diagram of relative abundance of Fusobacteria and Scr concentration; **(C)** the scatter diagram of relative abundance of Patescibacteria and Scr concentration.

The correlation between the predominant species of salivary microbiota in RTRs and ESRD and the corresponding Scr on the day of saliva collection was analyzed (Table 2 and Figure 4). In the RTRs group, Bacteroidetes, Fusobacteria, Patescibacteria, and Leptotrichiaceae were positively correlated with Scr, whereas Streptococcaceae was negatively correlated with Scr after renal transplantation. However, these floras were not significantly associated with Scr in the ESRD group.

**Table 2:** Pearson correlation between the salivary microbiota and Scr after renal transplantation.

	**RTR**		**ESRD**	
	**Pearson** ***r***	***P***	**Pearson** ***r***	***P***
p__Bacteroidetes	0.3329	0.0384	0.4095	0.2111
p__Fusobacteria	0.3436	0.0322	−0.3558	0.2829
p__Patescibacteria	0.704	<0.0001	0.2271	0.5019
c__Bacteroidia	0.3321	0.0389	0.4095	0.2111
c__Fusobacteriia	0.3436	0.0322	−0.3558	0.2829
o__Fusobacteriales	0.3436	0.0322	−0.3558	0.2829
f__Streptococcaceae	−0.3166	0.0496	−0.0437	0.8984
f__Leptotrichiaceae	0.3409	0.0337	0.4137	0.2059
g__Streptococcus	−0.3166	0.0496	−0.0437	0.8984
g__Leptotrichia	0.3411	0.0336	0.4475	0.1675

## 4. Discussion

Salivary microbiota is more stable than gut microbiota, and factors that alter gut microbiota may not significantly alter salivary microbiota (David et al., 2014; Tuganbaev et al., 2022). ESRD patients have changes in the composition of their gut microbiota compared with healthy people (Rysz et al., 2021; Shivani et al., 2022). However, as shown in this research, the salivary microbiota of ESRD patients was similar to that of HCs in terms of the number of individual species, the relative abundance of dominant flora, alpha diversity, and beta diversity, and did not alter significantly due to chronic renal impairment, different long-term treatments, or accompanying changes in life habits. In contrast, the salivary microbiota of RTRs showed huge differences compared with ESRD and HC groups. RTRs contained nearly 10 times as many species of unique salivary microbiota. From both alpha diversity and beta diversity, the RTR group showed higher richness and intra-group differences than ESRD or HC groups. At the phylum level, the relative abundance of Actinobacteria, Tenericutes, and Spirochaetes was about 10 times higher than that of ESRD or HC groups. Actinobacteria is a ubiquitous gram-positive phylum, which has attracted much attention as a rich source of bioactive substances and a complex evolution and diversification process (Miao and Davies, 2010; Barka et al., 2016). As an oral bacterium, Actinobacteria may play a role in the etiology of diabetes (Long et al., 2017; Matsha et al., 2020). The Tenericutes were composed of bacteria that lack a peptidoglycan cell wall. The most well-studied branch of this phylum was Mollicutes, including Mycoplasma. To date, most studies had focused on pathogenic strains of the Mycoplasma order (Wang Y. et al., 2020). As reported, Mycoplasma was associated with oral leukoplakia (Mizuki et al., 2015, 2017), mucositis (Morand and Hatami, 2018), and Fanconi anemia-associated oral carcinoma (Henrich et al., 2014). Spirochaetes were important pathogenic bacteria in the clinic, but they were not well-understood. Some of these caused Lyme disease, leptospirosis, syphilis, and other human diseases. Moreover, Spirochaetes were closely related to periodontal disease and gingivitis (Reed et al., 2018; Yousefi et al., 2020; Zeng et al., 2021), and, in turn, periodontal disease impacted the risk of systemic diseases such as diabetes (Deng et al., 2018). Taken together, these changes occurring in the salivary microbiota of RTRs appeared to be associated with the new onset diabetes, periodontal disease, and gingivitis after renal transplant.

During the early stage (<14 days) after surgery, the salivary microbiota of RTRs was not static. From the Venn graph, we could see that the total number of species decreased over time and most of the salivary microbiota were species unique at each time point. At the phylum level, the relative abundance of Actinobacteria, Cyanobacteria, Epsilonbacteraeota, Tenericutes, and Spirochaetes changed incrementally with time. Among them, Actinobacteria, Tenericutes, and Spirochaetes in RTRs had changed the most. From the point of alpha diversity, including kinds of evaluation indexes such as Ace, Chao1, Shannon, and Simpson, the composition of the salivary microbiota generally moved toward less richness and less variation within groups for each index. As shown in the PCoA figure, RTR1, RTR3, RTR7, and RTR14 groups all had different flora structures. However, the microbiota structure had significant differences only between RTR3 and RTR7 but not RTR1 and RTR3 or RTR7 and RTR14 which may be limited by the insufficient sample size. Moreover, the Chao1 and Ace, two alpha diversity evaluation indexes, described the distribution of bacteria with low abundances and decreased as the number of OTUs decreased during the perioperative period. Hence, we speculated that these low-abundance floras, which occurred in huge changes at different time points after renal transplant, occupy the majority of OTUs. As discussed, RTR14 was closer to HC in terms of OTU number and alpha diversity of salivary microbiota than RTR1. Hence, we considered that changes in the composition of the salivary microbiota of RTRs were a process of stabilization during the early stage after renal transplant.

All renal transplant patients received ATG preoperatively, a drug that inhibited thymocyte activity and helped patients fight off rejection. ATG was also often used to treat severe aplastic anemia and altered patients' salivary microbiota but did not lead to a clear change in diversity over time (Ames et al., 2019). Similarly, RTRs were also all treated with FK506 and MMF which were related to oral cancer (Li et al., 2021) or oral ulcer (Asare and Gatzke, 2020), and these oral diseases were also related to salivary floras (Lin et al., 2021; Bai et al., 2022). Not only salivary microbiota but these immunosuppressants had been linked to the altered intestinal microbiota. Intaking a moderate dose of FK506 maintained immunosuppression, induced normal graft function of the liver, maintained gut barrier integrity, and low plasma endotoxin levels. In addition, it also led to increased species richness and rare species abundance which was consistent with our findings (Jiang et al., 2018). FK506 treatment significantly improved the relative abundance of Bacteroides (Zhang et al., 2018). In our study, the relative abundance of Bacteroides decreased than increased. As an ongoing drug, the effect of FK506 on the elevating relative abundance of Bacteroides may not manifest until the latter part of the early stage after kidney transplantation. MMF enhanced colonic integrity and decreased sympathetic drive in the gut which was associated with the improvement of gut dysbiosis, including the increased abundance of Proteobacteria and Bacteroidetes and decreased abundance of Firmicutes (Robles-Vera et al., 2021) such as the results of our study that the abundance of Firmicutes continuously reduced. MMF increased the alpha diversity of gut microbiota embodied in the first postoperative day of our research (Llorenç et al., 2022). Based on the induction therapy and immunosuppressive therapy, RTRs had immune dysfunction such as acquired immunodeficiency disease (AIDS) patients, according to which we speculated that salivary microbiota changes were similar to those in AIDS patients. In the research of Perez Rosero et al., the significant reduction in the frequency of oral neutrophils in the oral cavity of AIDS individuals was positively related to their CD4+ T cell count and observed OTUs indexes raised in AIDS individuals as alpha diversity of salivary microbiota (Perez Rosero et al., 2021). Interestingly, Alpha diversity altered as the disease progresses (Guo et al., 2021). Compared with healthy people, AIDS patients exhibited a lower abundance of salivary Fusobacteria resembles our study (Yang et al., 2020). For AIDS patients, antiretroviral therapy was an effective treatment. After the treatment, the patient's immune function would be restored to some extent, which was due to the changes in immune function during the perioperative period in RTRs as the gradual recovery of T cell abundance occurred (Bouteloup et al., 2017). These two statuses were all accompanied by decreased salivary alpha diversity (Imahashi et al., 2021). Although RTRs were essentially on constant antibiotics, previous studies had demonstrated that antibiotic use appears to have little effect on salivary flora composition (Tuganbaev et al., 2022). Coincidentally, some external factors which may affect salivary microbiota for patients, such as diet habits (Marsh et al., 2016), drinking water (Sinha et al., 2021), oral hygiene (Belstrøm et al., 2018b), and living environment (David et al., 2014), changed between these time points of saliva collection. In conclusion, we speculated that the factors mentioned earlier functioned together and led to the alternation of salivary microbiota in RTRs like increasing and then gradually decreasing the number of OTUs and alpha diversity index and changing the composition of species and relative abundance of dominant flora with various trends.

Finally, we analyzed the relationship between the dominant flora in saliva and Scr. We found that Bacteroidetes, Fusobacteria, Patescibacteria, and Leptotrichiaceae were positively correlated with Scr, and Streptococcaceae was negatively correlated with Scr after renal transplant. Therefore, these strains could be biomarkers of postoperative recovery of RTRs.

Although the presence of the floras Bacteroidetes, Fusobacteria, Patescibacteria, Leptotrichiaceae, and Streptococcaceae in saliva and their potential correlation with renal function have barely been researched, several studies have elucidated the relation of some of them in the gut and renal dysfunction. Studies have shown an increase in the relative abundance of gut Bacteroidetes in patients with stage 4–5 chronic kidney disease or patients with ESRD receiving hemodialysis (Crespo-Salgado et al., 2016; Wu et al., 2021). Although urinary stones are unlikely to cause kidney damage, urolithiasis patients had significantly lower microbial abundance and higher proportions of Bacteroidetes (Zhou et al., 2020). In a study by Li et al., uremic clearance granules enhanced renal function and decreased levels of Scr, blood urea nitrogen, inflammatory responses, and NF-κB and MAPK expressions in renal tissues of ESRD rats. At the same time, the relative abundances of gut Bacteroidetes descended in response to uremic clearance granules (Li et al., 2022). As a prescription of traditional Chinese medicine for treating chronic kidney disease, the Shenyan Kangfu tablet alleviated renal dysfunction, glomerular and tubular damage, and renal inflammation and reduced the relative abundances of gut Bacteroidetes in the mouse with diabetic kidney disease (Chen et al., 2021). Accompanied by the fecal microbiota transplant, a significant increase of gut Bacteroidetes had the closest correlation with worse response to high salt of salt-sensitive rats, evidenced by increased albuminuria, systolic arterial pressure, and renal T-cell infiltration (Abais-Battad et al., 2021). By contrast, SGL5213 and Daphnetin, two proven renoprotectants, saved kidney function in mice or rats with renal injury and elevated the relative abundances of gut Bacteroidetes (Ho et al., 2021; Zhou et al., 2022).

The relative abundance of Fusobacteria in patients with immunoglobulin A nephropathy or membranous nephropathy exhibited significant elevation when compared with healthy controls (Hu et al., 2020; Zhang et al., 2020; Sugurmar et al., 2021). The microbiota structure showed the same change in type 2 diabetes mellitus, chronic kidney disease, and renal uric acid stone patients (Salguero et al., 2019; Cao et al., 2022). Deltamethrin, as a widely used pyrethroid insecticide, had brought serious problems to the healthy breeding of aquatic animals. A high concentration of deltamethrin damaged the intestine and trunk kidney of goldfish or channel catfish in the early stage with a significant increase or decrease in the abundance of Fusobacteria (Zhou et al., 2021; Yang et al., 2022).

In summary, gastrointestinal Bacteroidetes and Fusobacteria in humans and mice were positively correlated with renal dysfunction which was consistent with our results. Hence, we speculated that these two floras and even more flora may have some connection with renal dysfunction. However, whether the changes in the digestive tract environment brought by renal dysfunction favored their colonization of the digestive tract or their colonization of the digestive tract promoted renal dysfunction remained to be proven.

## 5. Conclusion

This study has illustrated differences in salivary microbiota communities among RTRs, ESRD patients, and HCs, examining changes in the salivary microbiota community during the short period after renal transplantation. We speculated that changes in the salivary microbiota were a process of stabilization during the early stage after renal transplant, and certain species of salivary microbiota had the potential to be a biomarker of postoperative recovery. Our study first discussed the salivary microbiota variations associated with renal transplantation and the relationship between salivary microbiota and renal function.

## Data availability statement

The data presented in the study are deposited in the NCBI repository, accession number PRJNA904953, https://www.ncbi.nlm.nih.gov/bioproject/PRJNA904953.

## Ethics statement

The study protocol was approved (22207) by the Ethics Committee of the Third Xiangya Hospital of Central South University (Changsha, China). Written informed consent was obtained from all study participants. Experiments were carried out in accordance with the ethical guidelines set by the Declaration of Helsinki 1964 and its later amendments. The patients/participants provided their written informed consent to participate in this study.

## Author contributions

XX, BP, YZ, and YM conceived, designed, and directed the manuscript. XX, BP, and KL wrote and revised the manuscript. XX, PD, and HL participated in the performance of the research. XX, TW, and BP analyzed data. All authors contributed to the article and approved the submitted version.
