# Supplementary figures and images for: Association between salivary microbiota and renal function in renal transplant patients during the perioperative period
Source: Front Microbiol. 2023 Mar 29;14:1122101. doi: 10.3389/fmicb.2023.1122101 (PMC10090686; doi:10.3389/fmicb.2023.1122101)

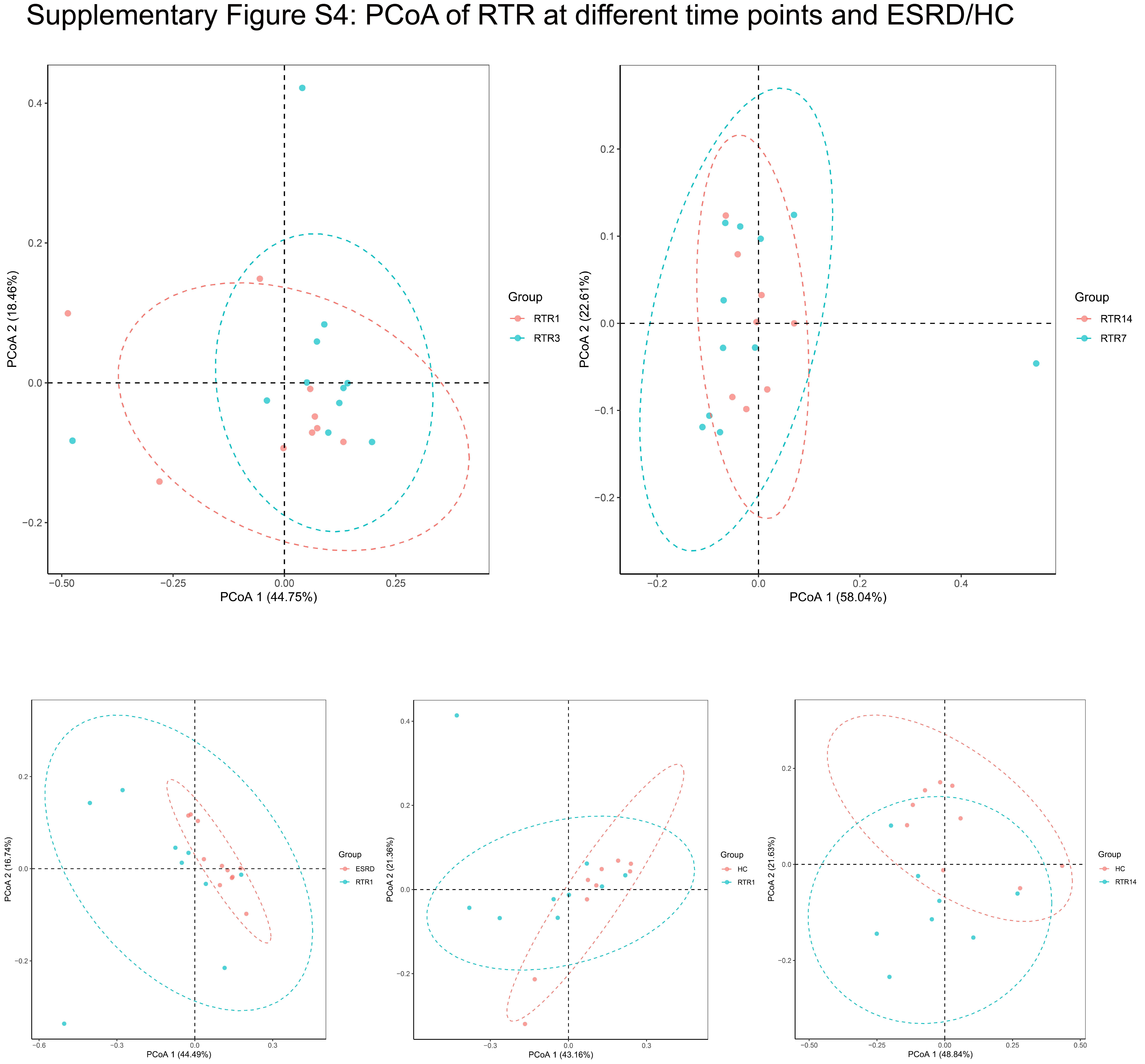

Supplement: Supplementary file 1 [file Data_Sheet_1.ZIP › Supplementary Figure S4.jpg]
